# The Importance of Gender-Stratified Antibiotic Resistance Surveillance of Unselected Uropathogens: A Dutch Nationwide Extramural Surveillance Study

**DOI:** 10.1371/journal.pone.0060497

**Published:** 2013-03-29

**Authors:** Casper D. J. den Heijer, John Penders, Gé A. Donker, Cathrien A. Bruggeman, Ellen E. Stobberingh

**Affiliations:** 1 Department of Medical Microbiology, Maastricht University Medical Centre/Care and Public Health Research Institute, Maastricht, The Netherlands; 2 NIVEL, The Netherlands Institute for Health Services Research, Utrecht, The Netherlands; Northwestern University, United States of America

## Abstract

Few studies have been performed on urinary tract infections (UTIs) in men. In the present study, general practitioners (n = 42) from the Dutch Sentinel General Practice Network collected urinary samples from 560 male patients (≥18 years) suspected of UTI and recorded prescribed antibiotic treatment. In this way, the antibiotic susceptibility of Gram-negative uropathogens, including extended-spectrum beta-lactamase (ESBL-) producing *Escherichia coli* could be determined. In addition, *E. coli* susceptibility and antibiotic prescriptions were compared with data from a similar UTI study among women and with data collected 7 years earlier. Of 367 uropathogens (66%) identified (≥10^3^ cfu/mL), most were Gram-negative (83%) and *E. coli* being isolated most frequently (51%). Antibiotic susceptibility to ciprofloxacin, norfloxacin and nitrofurantoin was 94%, 92% and 88%, respectively, whereas co-amoxiclav (76%) and co-trimoxazole (80%) showed lower susceptibilities. One ESBL (0.5%) was found. A significantly higher proportion of female UTIs was caused by *E. coli* compared with men (72% versus 51%, P<0.05). *E. coli* susceptibility tended to be lower in men compared with women, although not reaching statistical significance. No changes in *E. coli* susceptibility were observed over time (all P>0.05). Co-amoxiclav and nitrofurantoin prescriptions increased over time (11% versus 28% and 16% versus 23% respectively, both P<0.05), whereas co-trimoxazole prescriptions decreased (24% versus 14%, P<0.05). In conclusion, given the observed gender differences in uropathogen distribution and (tendency in) *E. coli* antibiotic susceptibility, empirical male UTI treatment options should be based on surveillance studies including men only. When awaiting the culture result is clinically not possible, fluoroquinolones are advised as first-choice antibiotics for male UTIs in Dutch general practices based on current antibiotic susceptibility data. The prevalence of ESBL-producers was low and no differences were observed in antibiotic susceptibility over a 7-year period. In addition, antibiotic prescriptions changed in accordance with national guidelines during this time period.

## Introduction

Most studies of urinary tract infections (UTIs) focus on female patients because of the higher incidence in women than in men. For this reason, most UTI guidelines are based on studies performed among women, despite the obvious genito-urinary differences [1]. Even so, it was estimated that in 2010 128,000 episodes of UTI were experienced by men in the Dutch general practice setting, representing 16 per 1000 male patients [2].

Several authors have stressed the importance that gender-stratified UTI surveillance studies should be performed, because of observed differences in antimicrobial susceptibility [3], [4]. However, these studies were biased by the inclusion of selected isolates, probably after initial treatment failure leading to an overestimation of resistance [3], [5].

In the Netherlands, regular surveillance studies are performed on unselected urinary isolates of both men and women to evaluate empirical treatment options in general practice, from which the female data have recently been reported [6]. With this data, we were able to assess the actual difference in *E. coli* susceptibility between male and female patients.

Furthermore, the current treatment options for male UTIs could be evaluated as stated in the guidelines of the Dutch College of General Practitioners (NHG). These are based on the presence of symptoms of tissue invasion (i.e. high fever (>38°C), chills, malaise, flank or perineal pain) with nitrofurantoin as first-choice agent and trimethoprim as second option for men without these symptoms. Male UTI with symptoms of tissue invasion should preferably be treated with co-amoxiclav followed by fluoroquinolones and co-trimoxazole as shared second-choice [7].

Also, the prevalence of extended-spectrum beta-lactamase (ESBL-) producing *E. coli* was assessed, as male gender has been identified as a risk factor for ESBL-producing Enterobacteriaceae [8].

Finally, differences in antibiotic susceptibility results over time could be determined for men, because a similar UTI surveillance study was performed between January 2003 and December 2004 [9].

## Methods

### Ethics statement

All data in this study were analyzed anonymously, only diagnostic specimens sent for diagnostic investigations and clinical data were used. Therefore, no consent was required from the patient and the ethics committee did not have to be approached. This is in agreement with the Medical Research Involving Human Subjects Act, the code for proper use of human tissue as formulated by the Dutch Federation of Medical Scientific Societies and the policy of the Medical Ethics Committee of the Maastricht University Medical Centre.

### Patients and antibiotic prescriptions

General practices (n = 42) from the Dutch NIVEL Sentinel General Practice Network were used for the recruitment of patients. The patient population of this network is nationally representative by age, gender, region and population density [10].

From January 2009 to June 2011, GPs included male general practice patients (≥18 years of age) with symptoms of UTI, i.e. dysuria, urinary frequency and/or urgency. Catheterized patients and patients suspected of having a sexually transmitted disease were excluded. With the exception of benign prostate hypertrophy, patients were also excluded when having urological or nephrological co-morbidity, diabetes mellitus or other immunocompromising diseases.

For all eligible patients, GPs filled in a patient form including age, symptoms of tissue invasion (fever (>38°C), flank pain) (yes/no) and empirical antibiotic treatment prescribed during the patient's visit (yes/no and if yes the prescribed agent was specified).

### Urine collection and processing

Patients provided a fresh-voided (midstream) urine sample, which was used to prepare a dipslide (Uriline, 56508, Biomérieux, Plainview, NY, USA) according to the manufacturer's instructions. For incubation and further microbiological analysis, dipslides were sent to the microbiological laboratory of Maastricht University Medical Centre, The Netherlands.

Bacterial growth was determined on the day of arrival and was considered positive at ≥10^3^ cfu/mL [11]. If no growth was observed, dipslides were incubated overnight at 37°C and assessed again. Standard microbiological methods were used for the identification of the uropathogens [12]. When more than one bacterium was cultured, only the predominant one was included in the final analysis. After isolation and identification, all bacterial strains were kept at −20°C in peptone/glycerol (30% w/v) for further testing.

### Antibiotic susceptibility testing

The susceptibility of Gram-negative isolates was determined using the microdilution method for the following antibiotics: amoxicillin, co-amoxiclav, trimethoprim, co-trimoxazole, norfloxacin, ciprofloxacin and nitrofurantoin. *E. coli* ATCC 35218 and ATCC 25922 were used as control strains. Methods and susceptibility breakpoints were in accordance with the EUCAST guidelines [13].

Putative ESBL-producing *E. coli* strains were selected on the basis of resistance to co-amoxiclav [6], and confirmed by means of a combination disc diffusion test (Neo Sensitabs, Rosco Diagnostica, Denmark) with ceftazidime, cefepime and cefotaxime with and without clavulanic acid, following the guidelines of the Dutch Society of Medical Microbiology (NVMM) [14].

### Comparison of E. coli susceptibility between men and women

From January 2009 to July 2009, a similar surveillance study among women suspected of UTI was performed [6]. This study was conducted by the same research group as the present study and patients were also recruited by GPs from the Dutch NIVEL Sentinel General Practice Network.

### Comparison between 2004 and 2011 data

The chosen categories (uropathogens and antibiotics) and all methods, including the network of the participating GPs, were the same in both studies, except for the guidelines used for the *E. coli* susceptibility breakpoints, i.e. CLSI (2004) and EUCAST (2011). Since both studies were performed by the same research group, the original MIC values from the 2004 study were available and 2004 susceptibility data could be recalculated according to the EUCAST guidelines.

### Statistical methods

For the comparison of two groups, a Pearson's Chi-square test was used for categorical variables and an independent-samples t-test for continuous variables when parametric assumptions were met. The Mantel-Haenszel extension test for trend was used to determine a trend between more than two categorical variables. The programme PSAW 18.0 for Windows was used for statistical analyses and P<0.05 was considered statistically significant.

## Results

### Patients and culture results

In total, 603 men were included with a median age of 65 years (range 18–97 years). Patients were considered eligible for analyses when data from the patient form were complete (560/603, 93%). The median age of population with complete data did not differ from the full patient population. Of these 560 patients, 137 (24%) had symptoms of tissue invasion. The median age of the populations with and without signs of tissue invasion was similar (66 years versus 64 years, respectively, P>0.05).

A positive culture result was found in 367 (66%) men, of whom 100 (27%) had symptoms of tissue invasion. The probability of a culture being positive increased with age, with men aged 70+ having a 3-times higher chance of a positive culture than men below the age of 50 years (Table 1).

**Table 1:** Relationship between patient's age and culture result.

	Culture result^a^			
Age categories (years)	Bacteriuria^b^ (n = 367)	No bacteriuria^b^ (n = 193)	OR (95% CI)	P for trend^c^
18–50	58	56	1.0 (reference)	
51–70	152	87	1.9 (1.2–2.9)	<0.001
>70	157	44	3.8 (2.3–6.2)	

**NOTE.** OR = odds ratio; 95% CI = 95% confidence interval. Values are given in numbers.

a Culture results per age category are given in numbers.

b Bacteriuria is defined as the presence of ≥10^3^ cfu/mL on the urine dipslide.

c P for trend was calculated using the Mantel extension test for trend.

Of all isolated uropathogens, 83% was Gram-negative with *E. coli* being most commonly found (51% of all uropathogens). Differences in uropathogen distribution per age category were not observed (Table 2).

**Table 2:** Distribution of isolated uropathogens per age category.

	Age category (years)			
	18–50	51–70	>70	Total
	(n = 58)	(n = 152)	(n = 157)	(n = 367)
*E. coli*	57	52	48	51
*P. mirabilis*	3	3	8	5
Klebsiella species	12	5	4	6
Non-fermenters^a^	10	9	11	10
Other Gram-negatives^b^	7	8	15	11
*Enterococcus species*	3	6	4	5
Other Gram-positives^c^	7	17	10	12

**NOTE.** Values are given in percentages.

a Consist of Pseudomonas and Acinetobacter species.

b Consist of Morganella, Citrobacter, Serratia, Pasteurella, Providentia and Enterobacter species.

c Consist of *Staphylococcus saprophyticus, Staphylococcus aureus* and Streptococcus species

No trends with age were observed for the given uropathogens (all P>0.05).

### Prevalence of antibiotic susceptibility

With respect to the preferred agents for men without signs of tissue invasion according to NHG, Gram-negatives showed a high overall susceptibility to nitrofurantoin (88%), whereas a lower susceptibility was found to trimethoprim (75%). Co-amoxiclav, the first-choice agent for men with signs of tissue invasion according to NHG, showed a relatively low antibiotic susceptibility (76%), as well as co-trimoxazole (80%). On the other hand, the fluoroquinolones (i.e. norfloxacin and ciprofloxacin) had a high susceptibility to the Gram negatives (92% and 94% respectively) (Table 3). When these Gram-negative susceptibility data were stratified according to the age categories mentioned earlier, i.e. 18–50, 51–70 and 70+ years, no differences were observed per antibiotic with age (all P>0.05, data not shown).

**Table 3:** Antibiotic susceptibility of Gram-negative uropathogens.

	Total (n)	Antibiotic susceptibility (%)						
Uropathogens		AMOX	AMC	TMP	SXT	NOR	CIP	NIT
*Escherichia coli*	188	65	84	77	78	93	94	100
*Proteus mirabilis*	18	82	100	82	88	100	100	41
Klebsiella species	21	0	83	94	100	100	100	94
Non-fermenters^a^	38	42	52	35	38	81	84	55
Other Gram-negatives^b^	39	20	49	83	89	89	97	86
**All Gram-negative uropathogens**	304	53	76	75	80	92	94	88

**NOTE.** AMOX  =  amoxicillin; AMC  =  co-amoxiclav; TMP  =  trimethoprim; SXT  =  co-trimoxazole; NOR  =  norfloxacin; CIP  =  ciprofloxacin; NIT  =  nitrofurantoin.^a^Consist of Pseudomonas and Acinetobacter species.^b^Consist of Morganella, Citrobacter, Serratia, Pasteurella, Providentia and Enterobacter species.

### Antibiotic use

Empirical antibiotic treatment was prescribed to 359 patients (64%), of whom 284 (79%) were later confirmed as being culture positive. 70+ year old men had a 2-times higher chance to receive antibiotic treatment empirically compared with 18-50 year old men (OR: 2.0, 95% confidence interval: 1.2–3.2, P for trend <0.001). Fluoroquinolones showed the highest prevalence of prescription (29%), followed by co-amoxiclav (28%), nitrofurantoin (23%) and co-trimoxazole (14%).

**Figure 1:**
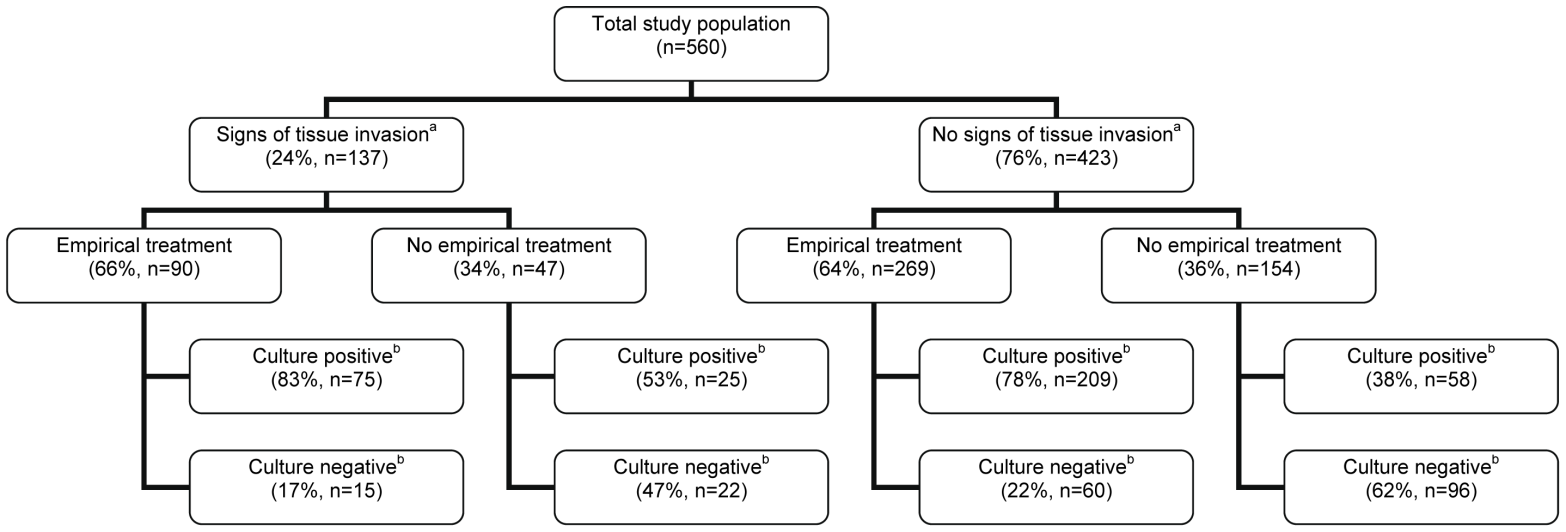
Patient population flow diagram based on signs of tissue invasion, empirical therapy and culture result. The denominator of the given percentage per box was derived from the number given in the box one level up.^a^Consisted of fever (>38°C) and flank pain.^b^Based on the presence of ≥10^3^ cfu/mL uropathogens on the urine dipslide.

In Figure 1 a flow chart is given based on the presence or absence of signs of tissue invasion, empirical therapy and a positive culture result. In the group with signs of tissue invasion (n = 137), co-amoxiclav was prescribed more frequently and nitrofurantoin less frequently compared with the prescriptions of the population without signs of tissue invasion (39% versus 24%, P = 0.004, and 11% versus 27%, P = 0.002, respectively).

### Comparison of E. coli susceptibility between men and women

In women, *E. coli* accounted for 72% of the total isolates, compared with 51% in men (P<0.001). Except for nitrofurantoin, *E. coli* susceptibilities tended to be lower in men compared with women: amoxicillin (65% versus 66%), co-amoxiclav (84% versus 87%), trimethoprim (77% versus 81%), co-trimoxazole (78% versus 84%), fluoroquinolones (94% versus 97%) and nitrofurantoin (both 100%), without being significant for the antibiotics tested (all P>0.05).

### Comparison of 2004 and 2011 data

No differences were observed between *E. coli* antibiotic susceptibilities in the 2004 and 2011 studies (Table 4). This also applied to the prevalence of extended-spectrum beta-lactamases (ESBLs), as no ESBL-producing *E. coli* were found in 2004 and one (0.5%) in 2011 (P = 0.44).

**Table 4:** *Escherichia coli* antibiotic susceptibility between 2004 and 2011 studies according to guidelines used for susceptibility breakpoints.

	Study year		
	2004 (n = 113)		2011 (n = 188)
	CLSI	EUCAST	EUCAST
Amoxicillin	75	72	65
Co-amoxiclav	100	89	84
Trimethoprim	81	78	77
Co-trimoxazole	81	81	78
Norfloxacin	97	95	93
Ciprofloxacin	97	96	94
Nitrofurantoin	97	97	100

**NOTE.** Values are given in percentages.

No significant differences in *E. coli* susceptiblilities (EUCAST) were observed between 2004 and 2011 study (all P>0.05)

No significant differences in *E. coli* susceptibilities (EUCAST) were observed between 2004 and 2011 study (all P>0.05)When comparing the antibiotic prescriptions between 2004 and 2011, GPs prescribed more co-amoxiclav and nitrofurantoin (11% versus 28%, P<0.001, and 16% versus 23%, P = 0.023, respectively) and less co-trimoxazole (24% versus 14%, P = 0.002) in 2011. For fluoroquinolones, amoxicillin and trimethoprim the prevalence of prescription was similar in the two studies (33% versus 29%, 2% versus 3% and 2% versus 1% respectively).

## Discussion

In this study of UTIs in Dutch general practice, differences were observed between men and women regarding the distribution of uropathogens, and uropathogenic *E. coli* susceptibilities tended to be lower among men compared with women. For male UTIs, fluoroquinolones showed the highest prevalence of susceptibility to isolated Gram-negative uropathogens, followed by nitrofurantoin. ESBL-producing *E. coli* prevalence was low and no differences in *E. coli* susceptibility were observed over a 7-year period.

The important feature of this study is the participation of general practices from the NIVEL Sentinel General Practice Network. This network has a nationally representative patient population, making it possible to generalize our conclusions to the whole Dutch outpatient population. Moreover, to our knowledge, no other study has been able to include over 600 unselected urinary samples from men suspected of UTI in an outpatient setting.

The fact that no susceptibilities were determined for Gram-positive bacteria could be seen as a limitation. However, empirical treatment for uncomplicated female UTIs is often primarily based on *E. coli* susceptibilities, covering 75–95% of the total spectrum of uropathogens in female UTIs [15]. The proportion of Gram-negatives in the present study is within this range, as also described by Lipski [16], thereby supporting an evidence-based empirical antibiotic choice for UTI in men.

Also, our study population could be regarded as heterogeneous with the inclusion of men aged 18+, because pathophysiology of male UTI differs with age [16]. We have tried to circumvent this problem by using strict in- and exclusion criteria, in order to obtain a homogeneous sample of men suspected of UTI. Moreover, stratification of antibiotic susceptibility results showed no differences by age.

UTI in men has also been studied by Hummers-Pradier et al. and they used 10^2^ and 10^5^ cfu/mL as cut-off values to determine a UTI [17]. However, their prevalence of UTI (60%), using 10^2^ cfu/mL as cut-off, was still lower than our prevalence. The fact that only 36% of the patients included were prescribed an antibiotic in that study and that GPs suspected a UTI in just over 50% of the included patients, suggests that their patient population was at lower risk of UTI than our population with a prescription rate of 64%. In addition, their results were derived from a relatively small patient population (n = 79), resulting in estimates with large confidence intervals. Our choice to use 10^3^ cfu/mL as cut-off value for the diagnosis of male UTI was based on the European urinalysis guidelines and is supported by Lipsky et al. [11], [18].

The prevalence of positive culture (66%) we found was also slightly higher than the one reported in the 2004 study (56%) [9]. The higher median age in the present study (65 versus 58 years) could explain this difference, because it is known that UTI incidence increases with age, and especially in men, due to the higher frequency of prostatic hypertrophy [16]. In the present study we could confirm this age-dependent trend for positive cultures.

Only 51% of the uropathogens causing male UTI was *E. coli* versus 72% of the UTIs in women. Moreover, uropathogenic *E. coli* susceptibilities tended to be lower in men in women, although no statistical significance was reached. The difference in these important characteristics for determining optimal empirical treatment, show that men with UTI should be considered as a specific patient category.

Given the more heterogeneous population of uropathogens that causes a UTI in men compared with women, empirical treatment should best be avoided for male UTI. When immediate treatment is necessary based on clinical judgement, our data can be used for the optimal choice of empirical treatment. Our recommendations can be applied to all men aged 18+ suspected of UTI in the Dutch GP setting, because no differences in uropathogen distribution and antibiotic susceptibility per age category were observed.

We found that fluoroquinolones showed a higher prevalence of susceptibility than co-amoxiclav, the current first-choice for men with symptoms of tissue invasion [7], and the former agents possess specific pharmacokinetic properties leading to high prostate tissue concentrations, which co-amoxiclav lacks [19]–[21].

The choice for EUCAST instead of CLSI guidelines had an impact on the *E. coli* susceptibility to co-amoxiclav. Using EUCAST breakpoints, the *E. coli* susceptibility to co-amoxiclav in 2004 decreased from 100% to 89%. The current prevalence of susceptibility of Gram-negatives to co-amoxiclav (77%) is below the proposed threshold (80%) for antibiotics to still be effective as empirical treatment for UTI [22].

Based on these findings, fluoroquinolones may be regarded as the current preferred treatment in men with tissue invasion. However, in addition to antibiotic resistance data, side effects of the drugs related to age, financial consequences and patient characteristics should be included in the choice of the preferred antibiotics.

Except for *Proteus* species and non-fermenters, high antibiotic susceptibilities (≥87%) to Gram-negatives were also found for nitrofurantoin. Similar results were described by Guay [23]. Despite this high prevalence of susceptibility, clinical trials on the effectiveness of nitrofurantoin for male UTIs are currently lacking. Since nitrofurantoin does not reach therapeutic concentrations in prostatic tissue [21], differentiation between cystitis with and without prostatitis is important. So far, no symptoms have been found that allow a conclusive differentiation between these two conditions [24]. The Dutch GP guidelines have based their treatment choices on the presence or absence of so-called ‘symptoms of tissue invasion’, although no reference was given. Results from future trials would support evidence-based treatment of UTI in men and could help to restrict the use of fluoroquinolones, thereby limiting the chance of the development of antibiotic resistance to this antibiotic group. In this perspective, also the value of fosfomycin for male UTIs needs exploration [25].

For male UTIs, second-choice agents (i.e. fluoroquinolones) were still prescribed most, although overall compliance with national guidelines improved in comparison with prescription data from 2004. This was shown by an increase in co-amoxiclav and nitrofurantoin prescriptions, the first-choice agents for men with and without symptoms of tissue invasion respectively.

In the surveillance study on female UTIs in Dutch general practices, *E. coli* susceptibilities from 2004 and 2009 were compared, and also no differences in time trend were observed [6]. Several other studies, including urinary samples from men and women, have reported an increased resistance over time among isolates from an outpatient setting [26], [27]. Our regular surveillance studies, the results of which are implemented in clinical practice by including them in the update of the national GP guidelines, appear to have contributed to the control of antibiotic resistance in Dutch general practices. The low prevalence of ESBL-producing *E. coli* is in accordance with these findings.

In conclusion, among unselected *E. coli* originating from Dutch male GP patients suspected of UTI, the prevalence of ESBL-producers was low and no differences were observed in antibiotic susceptibility over a 7-year period. In addition, antibiotic prescriptions changed in accordance with national guidelines during this time period. For male UTIs in Dutch general practice, fluoroquinolones may be regarded as the best treatment option on the basis of current susceptibility data. Differences in uropathogen distribution, together with the tendency of a lower *E. coli* antibiotic susceptibility in men versus women, make it necessary to base male UTI treatment recommendations on UTI studies performed among men only.
